# Thiol–Ene Click Reaction Initiated Rapid Gelation of PEGDA/Silk Fibroin Hydrogels

**DOI:** 10.3390/polym11122102

**Published:** 2019-12-14

**Authors:** Jianwei Liang, Xiaoning Zhang, Zhenyu Chen, Shan Li, Chi Yan

**Affiliations:** State Key Laboratory of Silkworm Genome Biology, College of Biotechnology, Southwest University, Chongqing 400715, China

**Keywords:** silk fibroin, PEGDA, thiol–ene click reaction, gelation, drug delivery

## Abstract

In this work, poly(ethylene glycol) diacrylate (PEGDA) molecules were grafted to silk fibroin (SF) molecules via a thiol–ene click reaction under 405 nm UV illumination for the fabrication of a PEGDA/SF composite hydrogel. The composite hydrogels could be prepared in a short and controllable gelation time without the use of a photoinitiator. Features relevant to the drug delivery of the PEGDA/SF hydrogels were assessed, and the hydrogels were characterized by various techniques. The results showed that the prepared PEGDA/SF hydrogels demonstrated a good sustained-release performance with limited swelling behavior. It was found that a prior cooling step can improve the compressive strength of the hydrogels effectively. Additionally, the MTT assay indicated the prepared PEGDA/SF hydrogel is non-cytotoxic. Subcutaneous implantation of the PEGDA/SF hydrogel in Kunming mice did not induce an obvious inflammation, which revealed that the prepared PEGDA/SF hydrogel possessed good biocompatibility. Furthermore, the mechanism of the gelation process was discussed.

## 1. Introduction

It is well known that qualified biomaterials shall possess good mechanical properties and biological compatibility [1]. Silk fibroin (SF), the major protein in the silk produced by *Bombyx mori*, can meet above criteria. Silk fibroin is biodegradable [2] with minimal inflammatory reaction [3]. Therefore, silk fibroin has been widely used in the biomedical and clinical fields [4,5,6].

Hydrogels are three-dimensional hydrophilic networks, which are crosslinked via chemical and physical bonds, with the ability to absorb and retain large amounts of water without dissolution [7]. Many hydrogels, including natural and synthetic ones, were used as materials for drug delivery [8] and tissue engineering [9], as both drugs and cells could be encapsulated in the hydrogel matrices. Based on needs, silk fibroin can be made into different forms [10,11], and hydrogel is one of them. Although silk fibroin-based hydrogel has been widely investigated, its application is limited due to its poor mechanical properties [12,13], as well as its uncontrolled and long period of gelation time [14,15].

Poly(ethylene glycol) diacrylate (PEGDA) is a biologically inert material with particular use in tissue engineering and drug delivery [16,17]. PEGDA gels rapidly in the presence of a photoinitiator and UV irradiation at 365 nm at room temperature [18], which would be extremely useful for bringing them to an industrial scale or approaching them with a wide variety of applications [19], although many photoinitiators used are identified as hazards [20,21,22,23]. The initiator-free photopolymerization for PEGDA hydrogel formation also has been reported but is less common [24]. Although demonstrating excellent mechanical properties, PEGDA-only gels do not support cell attachment, as the gel surface inhibits the adsorption of adhesion protein, such as fibronectin; therefore, there is a lack of cellular attachment sites [25]. In addition, gelation at 365 nm UV light or near-UV radiation (with wavelengths of 200 to 400 nm) can cause pyrimidine dimer formation and other chemical changes in the DNA of cells [26].

Although it is reported that norbornene-functionalized SF can be incorporated in norbornene-functionalized 4-arm poly(ethylene glycol) (PEG4NB) through thiol–ene photocrosslinking [27], carbic anhydride used for norbornene functionalization is dangerous [28]. It would be desirable to prepare hydrogels in the absence of toxic ingredients, with a short and controlled gelation time, while keeping their good mechanical properties and biocompatibility. Therefore, we adopted a different strategy in which thiol groups were first installed onto silk fibroin molecules through carbodiimide chemistry. Since PEGDA contains two alkene groups attached to each end of the molecule, they can graft themselves onto SF molecules. Photoinitiators for thiol–ene reactions have been extensively used [29]. The usage of a photoinitiator could cause undesirable side reactions, such as surface oxidation or the removal of bound thiols [30]. In addition, it increases the cost of manufacture with tedious procedures. According to safety data sheets [31,32,33,34], most of the photoinitiators used for thiol-ene reactions are harmful. Through the developed approach, the gelation of PEGDA/SF aqueous solution could be completed within three minutes without the appearance of a photoinitiator. Furthermore, the light used to mediate the thiol–ene reaction has a wavelength of 405 nm. Therefore, we present here a mild and green strategy for SF-based hydrogel fabrication. The release kinetics of the prepared hydrogel were studied using rhodamine B as a model molecule in order to evaluate its potential use in drug delivery application.

## 2. Materials and Methods 

### 2.1. Materials

Cocoons of silkworm *Bombyx mori* (a Chinese strain demoted as 872) were provided by the College of Biotechnology, Southwest University. Tris(2-carboxyethyl) phosphine hydrochloride (TCEP·HCl), 2-Morpholinoethanesulfonic acid (MES), and sodium chloride were purchased from Aladdin Agent Co., Ltd. (Shanghai, China). Sodium carbonate, rhodamine B (RB), and sodium biphosphate dihydrate were purchased from KeLong Chemical Reagent Co., Ltd. (Chengdu, China). Sodium dihydrogen phosphate was purchased from Fangzheng reagent Co., Ltd. (Tianjin, China). *N*-hydroxysuccinimide (NHS) and reduced glutathione (GSH) were purchased from Solarbio Co., Ltd. (Beijing, China). 1-(3-Dimethylaminopropyl)-3-ethylcarbodiimide hydrochloride (EDC) was purchased from Adamas-beta (Shanghai, China). Calcium chloride was purchased from Yuanye Bio-Technology Co., Ltd. (Shanghai, China). Ethanol was purchased from Chuandong Chemical Co., Ltd. (Chongqing, China). Poly(ethylene glycol) diacrylate (PEGDA, n = approximately 4) was purchased from Tokyo Chemical Industry Co., Ltd. (Tokyo, Japan). Potassium bromide was purchased from Sangon Biotech Co., Ltd. (Shanghai, China). Dulbecco’s modified Eagles medium (DMEM, high glucose) and phosphate-buffered saline (PBS, 1×) were purchased from Hyclone (Logan, UT, USA). 3-(4,5-dimethythiazol-2-yl)-2,5-diphenyl tetrazolium bromide (MTT) and dimethyl sulfoxide (DMSO) were purchased from BioFroxx (Einhausen, Germany). Fetal bovine serum (FBS) was obtained from Zhejiang Tianhang Biotechnology Co., Ltd. (Zhejiang, China). Hematoxylin and eosin (H&E) were purchased from Nanchang Yulu Experimental Equipment Co., Ltd. (Nanchang, China). All chemicals are of analytical grade and used without further purification. De-ionized (DI) water was produced by a Milli-Q Direct-8 purification system (resistivity >18 MΩ cm, Molsheim, France) onsite and used in all experiments.

### 2.2. Extraction and Purification of Silk Fibroin

Regenerated silk fibroin (RSF) solution was prepared according to the previous protocol developed [35]. *Bombyx mori* cocoons were cut to small pieces, and the silkworm chrysalises were removed from the cocoons. Then, 20 g cocoons were boiled in 1 L of 0.02 M Na_2_CO_3_ solution for 30 min and rinsed with distilled water by a magnetic stirrer (Shanghai Sile Instrument, T09-15, Shanghai, China) for 20 min. The above step was repeated one more time to get degummed silk fibers. In order to obtain RSF solution, degummed silk fibers were immersed in CaCl_2_–enthanol–H_2_O solution (molar ratio = 1:2:8) at 70 °C until the silk fibers were dissolved completely. Subsequently, the solution was dialyzed against double-distilled water (DI H_2_O) for 72 h in order to remove the impurities. The pH of the dialyzed SF solution was adjusted to 6.0 in 0.1M MES solution (containing 0.5 M NaCl) for 24 h. Next, the insoluble impurities were filtered out through the surgical gauze and then centrifuged at 8000 rpm (30 min, 4 °C). Later, the carboxyl groups on SF molecules were activated by EDC/NHS (0.5 mg/mL of EDC with 0.7 mg/mL of NHS in MES buffer) for 15 min at room temperature. Then, GSH was added to a final concentration of 2 g/L in above mixture. The reaction was conducted at room temperature for 15 min in order to couple GSH to the SF molecules covalently. After the completion of GSH coupling, the solution was dialyzed against DI H_2_O for another 24 h to remove unbound peptide and chemical remains. The prepared GSH-modified SF (GSH-SF) solution was lyophilized and then stored in a vacuum desiccator over silica gel at room temperature for later usage.

### 2.3. Hydrogel Preparation

To prepare the hydrogel, the lyophilized SF solid was dissolved in DI H_2_O to make 10% (*w*/*v*) GSH-SF solution, and TCEP·HCl (with a final concentration of 10 mmol/L) was added into GSH-SF solution in order to cleave disulfides. Fifteen min later, 1 mL of the above mixture and 1 mL of PEGDA were mixed in a 5 mL centrifuge cube and stirred using a vortex oscillator (VORTEX-5, Kylin-Bell Lab Instruments Co., Ltd., Haimen, China) for 3 s. Then, 600 μL of above mixture were transferred into a cylindrical mold (12 mm inner diameter) and placed 3 cm below a 30 W light-emitting diode (LED) UV light (405 nm wavelength, Shenzhen YuXianDe Science and Technology Ltd., Shenzhen, China). Each sample was illuminated by UV light for a certain amount of time.

### 2.4. Compressive Test

The compressive mechanical properties were measured by a universal testing machine (Shanghai Xieqiang Instrument Technology Co., Ltd., Shanghai, China). The compression rate was set at 2 mm/min, and the machine was stopped when the strain attained 50%.

### 2.5. Swelling Study

The lyophilized PEGDA/SF hydrogels were immersed directly in phosphate-buffered saline (PBS, pH = 7.4) solution for 6 h at 37 °C. The swollen hydrogel was removed from PBS solution at a predetermined time point (t = 30 min, 1 h, 2 h, 3 h, 6 h). The excess liquid was removed by blotting with filter paper. Then, the hydrogels were weighted individually on an analytical balance (Shanghai Jingtian Electronic Instrument Co., Ltd., Shanghai, China), and the swollen ratio (SR) was determined by the following equation [36]:

(1) $$SR=\frac{W_{t}-W_{d}}{W_{d}}\times 100\%$$

where *W_t_* is the weight of the swollen hydrogel at time *t*, and *W_d_* is the weight of the lyophilized hydrogel.

### 2.6. Fourier Transform Infrared Spectroscopy (FTIR) Analysis

The lyophilized PEGDA/SF hydrogel was ground into powders and mixed with KBr (ratio = 1:100, *w/w*). The above mixture was pressed into pellets for FTIR (Nicolet iN10, Thermo Scientific, Waltham, MA, USA) analysis. Each sample was scanned 24 times from 400 cm^−1^. to 4000 cm^−1^ with a resolution of 4 cm^−1^.

### 2.7. Scanning Electron Microscope (SEM) Observation

The prepared hydrogel sample was cut through to make a cross-section of this sample. The resulting cross-section was lyophilized and sputter-coated (MTI Corporation, Richmond, VA, USA) with gold to improve the conductivity of the surface for SEM (Phenom Pro, Phenom-World) observation.

### 2.8. Drug Release Test

Rhodamine B (RB) was selected as a model molecule for drug release study. The sustain release performance of RB-loaded hydrogel was tested according to the reference [37], and the concentration was determined by a UV-vis spectrophotometer (T6, Beijing Puxi Analytic Instrument Ltd., China). Linear regression of the solution concentration (C) and absorbance (A) of RB solution with different concentrations at λ = 555 nm was performed to obtain a standard curvilinear equation: C = 0.08756A-0.0025, R^2^ = 0.99914 (Supplementary Material, Figure S1). RB was mixed with GSH-SF and PEGDA to reach a final concentration of 0.1 mg/mL before gelation. After gelation, the samples were placed in weighing bottles filled with 4 mL of PBS solution (pH = 7.4). The weighing bottles and samples were placed on a shaker (Taicang Experiment Apparatus Ltd., Taicang, China) at 37 °C, with a rotational speed of 40 rpm/min. Then, 4 mL of the release solution was taken out at certain time intervals, and the UV absorbance of that solution at 555 nm was measured. Then, 4 mL of fresh PBS solution was added to the weighing bottles to replace the withdrawn solution.

### 2.9. Cytotoxicity Assay

The cytotoxicity of the hydrogel against the human embryonic kidney (HEK) 293 cell line was evaluated in vitro using a 3-(4,5-dimethyldiazol-2-yl)-2,5-diphenyltetrazolium bromide (MTT) assay based on the ISO 10993-5 standard. First, the hydrogels were rinsed by PBS (pH = 7.4) solution three times and then sterilized with high-pressure steam for 2 h. After sterilization, the hydrogels were immersed in Dulbecco’s modified Eagles medium (DMEM) to equilibrate (0.2 g hydrogel/9 mL DMEM) for 72 h in an incubator at 37 °C to obtain the leaching liquor of the prepared hydrogel. Then, the leaching liquor was filtered through the 0.22 μm pore size membrane (Tianjin Jinteng Experimental Equipment Co., Ltd., China). Next, HEK 293 cells were seeded in a 96-well plate with a density of 2 × 10^4^ cells/well in 200 μL complete growth medium (90% DMEM, 10% FBS and 1% penicillin streptomycin) and incubated at 37 °C in a 5% CO_2_ atmosphere for 24 h. Then, the culture medium was removed and replaced with 100 μL of complete growth medium. Later, 10 μL of the leaching liquor, complete growth medium, and phenol solution (64 g/L) was added into each well as experimental groups, blank groups, and positive control groups respectively. After 24 h of incubation, 20 μL of the 5.0 mg/mL MTT solution (dissolved in PBS) was added into each well for a further 4 h of incubation. After the removal of culture medium, the cells in each well were lysed in 200 μL of dimethyl sulfoxide (DMSO) to dissolve the formazan precipitate. After shaking on a Belly Dancer with gentle agitation for 15 min, the optical density at the wavelength of 490 nm was measured using a microplate reader (Biotek Synergy H1, Winooski, VT, USA), and each sample was tested in five replicates.

### 2.10. Subcutaneous Implantation

To determine the biocompatibility of the PEGDA/SF hydrogel in vivo, the PEGDA/SF hydrogels (1.3 mm in diameter and 0.18 mm in height) were implanted in three healthy female Kunming mice (18–22 g, specific pathogen-free), which were purchased from Tengxin Biotechnology Co., Ltd., (Chongqing, China). All the hydrogels were rinsed with PBS solution (pH = 7.4) three times and then sterilized by high-pressure steam. Then, a 1 cm incision was created on the posterior dorsomedial skin of the mouse, and a small lateral subcutaneous pocket was prepared by scissor dissection subsequently. The hydrogel sample was implanted in the subcutaneous pocket, and the incised wound was sutured. All the mice were individually housed in a sterilized cage with filtered air. The mice were provided autoclaved food and water. After three days, the mice were euthanized, and the hydrogels were retrieved along with the surrounding tissues for further analysis.

### 2.11. Histological Analysis

After implantation, samples were carefully rinsed with saline for three times and fixed in formaldehyde solution (4%, *v*/*v*), embedded in paraffin, and then cut into 5 μm thick sections using a microtome (Leica RM2235, Wetzlar, Germany). Then, the sectioned slides were stained by H&E followed by instruction from the manufacturer and observed under an optical microscope (DM3000, Leica, Wetzlar, Germany). The wounds were evaluated for the inflammation and granulation tissue formation.

The animal experimental design and procedures were executed according to the protocols approved by the Laboratory Animal Ethics Committee of Southwest University (Ethic Permission Code: IACUC-20190117-15; Date of Approval: 17 January 2019). Animal care and use strictly followed the Regulations for the Administration of Affairs Concerning Experimental Animals, National Committee of Science and Technology of China (14 November 1988), and Instructive Notions with Respect to Caring for Laboratory Animals, Ministry of Science and Technology of China (30 September 2006).

## 3. Results

### 3.1. Preparation of Hydrogel

**Figure 1:**
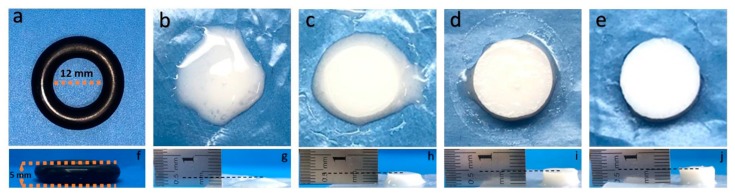
Photographs of mold used for sample preparation (**a**); samples after 30 s UV light illumination (**b**), 1 min of UV light illumination (**c**), 2 min of UV light illumination (**d**), and 3 min UV light illumination. (**e**) Figures (**f**–**j**) are the side views of Figure (**a**–**e**).

The photographs of the prepared PEGDA/SF hydrogels subjected to different amounts of UV light exposure are shown in Figure 1. The results indicated that the PEGDA/SF mixture remains in a liquid state after a 30 s UV light illumination (Figure 1b). Meanwhile, 1 min of UV illumination of the mixture could trigger the gelation, but the newly formed hydrogel was too soft to be handled by tweezers (Figure 1c). When the UV exposure time was elongated to 2 min, the hydrogel became more rigid to be held by tweezers, although there was still some liquid remains on the surface of the hydrogel (Figure 1d). After 3 min of illumination by UV light, the hydrogel surface was liquid-free, indicating that the gelation was complete (Figure 1e). Figure 1g–j presents the side views of the hydrogels. It can be observed that the height of the hydrogel increased with the increased amount of UV light exposure. After 3 min of UV light illumination, the hydrogel height reached more than 5 mm. Therefore, we could conclude based on our observations that the approach presented here can achieve a light-triggered local control of the hydrogel formation. Furthermore, the crosslinking density of the hydrogel could be tuned with UV exposure, and the stiffness of hydrogel increased due to the increasing of the crosslinking density.

### 3.2. Compressive Test Result

**Figure 2:**
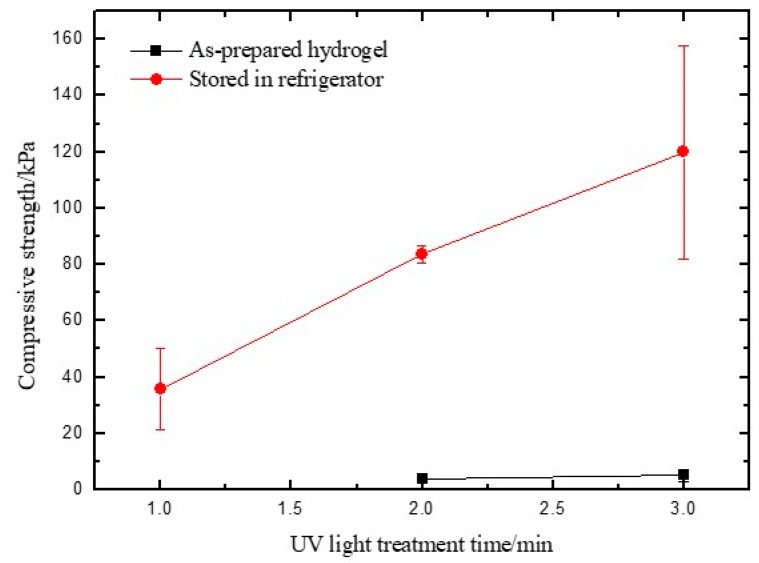
Compressive test results of as-prepared hydrogels and the hydrogels stored in a refrigerator at 4 °C for 24 h before testing. The data point for the sample illuminated by UV light for 1 min without a low-temperature treatment was missing, as the sample was too soft to be tested by the universal testing machine.

The mechanical properties of the prepared hydrogels were analyzed by a compressive test. It was found that the as-prepared hydrogel illuminated with UV light for 1 min cannot be tested by the universal testing machine, as it is too soft to be handled (Figure 1b). With an increase of UV light illumination time to 2 min, the formed hydrogel was strong enough to withstand shear force and be analyzed by a compressive test. It was found that the compressive strength of the composite hydrogel increased with increasing periods of time with UV light illumination (Figure 2), which is consistent with the observations during the preparation of hydrogels that indicated that the stiffness of the hydrogel increased with the increased amount of UV light exposure time.

As most hydrogels contain a variety of complex hydrogen bond networks across polymer chains [38,39], leveraging the hydrogen bond interaction can improve the compressive strength of the hydrogel [40]. In addition, it has been reported that low temperature played an important role in creating the stable hydrogen-bond networks through the formation of new hydrogen bonds [41]. Therefore, we stored one group of samples in a 4 °C refrigerator for 24 h before the compressive test. As Figure 2 demonstrated, the compressive strength of the as-prepared hydrogels illuminated with UV light for 2 min is of 3.81 ± 1.64 kPa. The compressive strength was increased to 83.73 ± 2.91 kPa after the samples were stored in 4 °C for 24 h. Similarly, the hydrogel prepared by 3 min of UV light illumination was reinforced by low-temperature treatment and obtained a 20-fold higher compressive strength. The results lead to the hypothesis that a prior cooling process can improve the hydrogen bond interactions, which could help interlock the PEGDA/SF hybrid and act to reinforce networks [42].

### 3.3. Swelling Ratio

**Figure 3:**
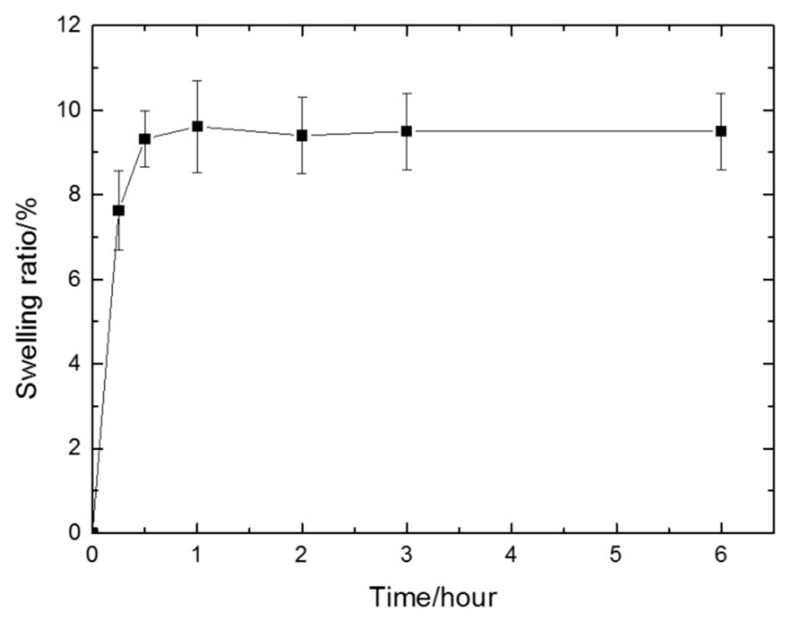
The swelling ratio of the prepared hydrogel in 6 h. After 1 h, the swelling ratio reached equilibrium (9.5%).

The extent to which the gel sample swells under the specific environmental condition can be defined as the swelling ratio. The swelling ratio is an important parameter to evaluate a hydrogel [43]. It possesses cumulative effects on the mechanical properties and can describe the diffusion and exchange of nutrients and waste through the entire hydrogel [44,45]. The result (Figure 3) showed that the swelling ratio increased dramatically in the first 30 min, indicating that the gels absorbed water rapidly in the early stage. Then, the crosslinking limited the penetration of water molecules into the hydrogel networks and restricted the extent of swelling. Therefore, the hydrogels tended to remain stable, reaching equilibrium after 1 h with a swelling ratio of 9.5%. The swelling ratio of the prepared PEGDA/SF hydrogel is lower compared with that of other SF-based hydrogels [46,47]. In general, the swelling behavior of a hydrogel is directly related to its stability; a hydrogel with a higher swelling ratio is prone to be less stable and is easier to be broken down or degraded [45,48].

### 3.4. Fourier Transform Infrared Spectroscopy Test Results

**Figure 4:**
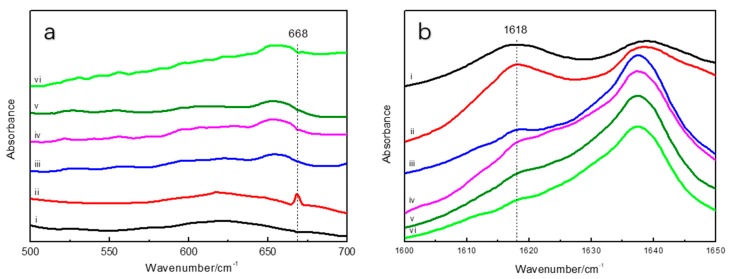
Fourier transform infrared (FTIR) spectra of silk fibroin (SF), reduced glutathione (GSH)-SF, and samples from 500 cm^−1^ to 700 cm^−1^ (**a**) and from 1600 cm^−1^ to 1650 cm^−1^ (**b**) i. SF, ii. GSH-SF, iii. sample after 30 s UV illumination, iv. sample after 1 min UV illumination, v. sample after 2 min UV illumination, vi. sample after 3 min UV illumination.

FTIR spectroscopy was used to characterize the molecular structure of the prepared hydrogel. The FTIR spectrum of the GSH-SF sample showed the characteristic S–H stretching vibration at 668 cm^−1^, which clearly indicated the presence of the thiol groups attached to the silk fibroin. Once the GSH-SF sample was mixed with PEGDA and illuminated with UV light for 30 s, the absorption peak at 668 cm^−1^ disappeared, because thiol groups interact with alkene moieties through a “thiol–ene” click reaction and generate a new chemical bond composed of S and C atoms (Figure 4a). The decrease in the intensity of band characteristics for the β-sheet at 1617 cm^−1^ indicated that the β-sheet content gradually reduced with the increase of UV light illumination time (Figure 4b). This might because the grafting of PEGDA onto silk fibroin interrupts the interstrand hydrogen bonds in the β-sheet, causing the change in the secondary structure of silk fibroin.

### 3.5. Scanning Electron Microscope

**Figure 5:**
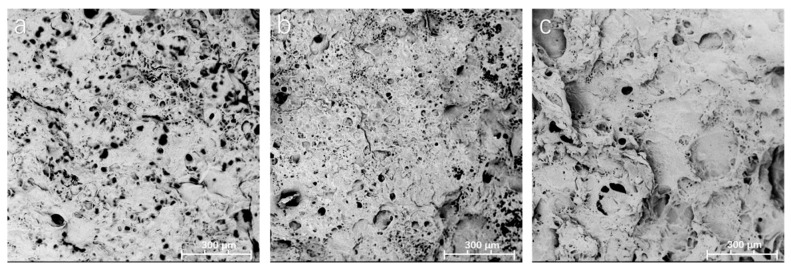
SEM images of hydrogel samples with UV light treatment for 1 min (**a**), 2 min (**b**), and 3 min (**c**).

The morphologies of lyophilized hydrogel samples were observed by scanning electron microscopy (SEM). Figure 5 shows the representative SEM images of the fractured cross-section of samples illuminated by UV light for 1 min, 2 min, and 3 min, respectively. All the samples have a porous structure. Although the geometry and the size of the pores are quite irregular, it could be found that the pore size was reduced as the UV light illumination time increased (Table 1). When taking into account that the “thiol–ene” click reaction was complete within 30 s of UV illumination and PEGDA can be photopolymerized under UV light in the absence of a photoinitiator [24] (Supplementary Material, Figure S2a), we hypothesized that UV illumination initiated the polymerization of PEGDA within the networks of the hydrogel, resulting in a higher network density, which could decrease the average pore size of the hydrogels. The pore size distribution was also provided in the Supplementary Material, Figure S3. Unlike that of the PEGDA hydrogel (Supplementary Material, Figure S2b), the exhibited porous structure of the developed PEGDA/SF hydrogel is expected to be useful for drugs or cells loading.

### 3.6. Drug Release

Rhodamine B (RB) was selected as a model molecule. The hydrogels were incubated in the PBS (pH 7.4) solution at 37 °C in order to mimic the in vivo environment. The cumulative amount of RB released was determined by a UV-visible spectrophotometer with an RB standard curve (Supplementary Material, Figure S1) according to the following equation [37]:

(2) $$Cumulative\;percentage\;of\;RB\;release=\frac{\sum_{1}^{n}vC_{n}}{m_{0}}\times 100\%$$

where *v* is the amount of release medium removed from the weighing bottle at a certain time (4 mL), *C_n_* is the concentration of *RB* released from hydrogels at the displacement time, *n* is the displacement time, and *m*_0_ is the total mass of *RB* contained in the hydrogel. Each experiment was performed in triplicate.

Simultaneously, the drug release kinetics of the prepared hydrogel was evaluated by fitting experimental data to Ritger–Peppas model [49]:

(3) $$\frac{M_{t}}{M_{\infty }}=k\;or\;ln\left(M_{t}∕M_{\infty }\right)=ln\;k+n\;ln\;t$$

where *t* is the release time, *M_t_* is the amount of drug released at time *t*, *M*_∞_ is the amount of drug released at an infinite time, *M_t_*/*M*_∞_ is the fraction of drug released at time *t*, *k* is a kinetic constant that depends on the construct of the structural and geometric characteristic of the hydrogel, and *n* is the diffusion exponent indicative of the mechanism of transport of drug through the hydrogel.

**Figure 6:**
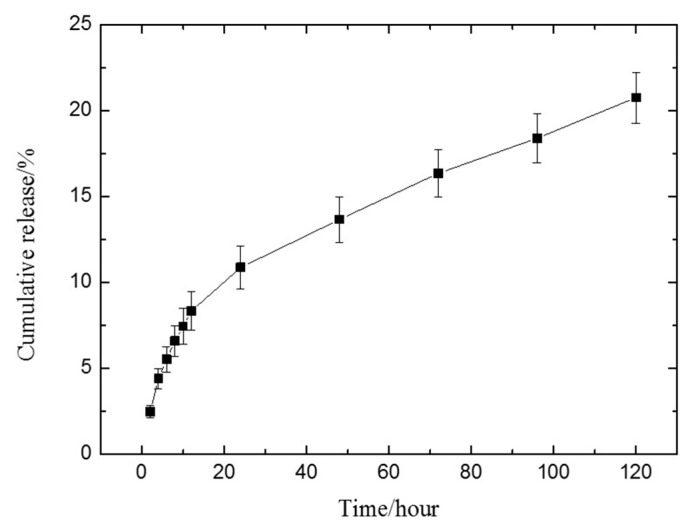
The release profiles of rhodamine B (RB) from the prepared hydrogel.

**Figure 7:**
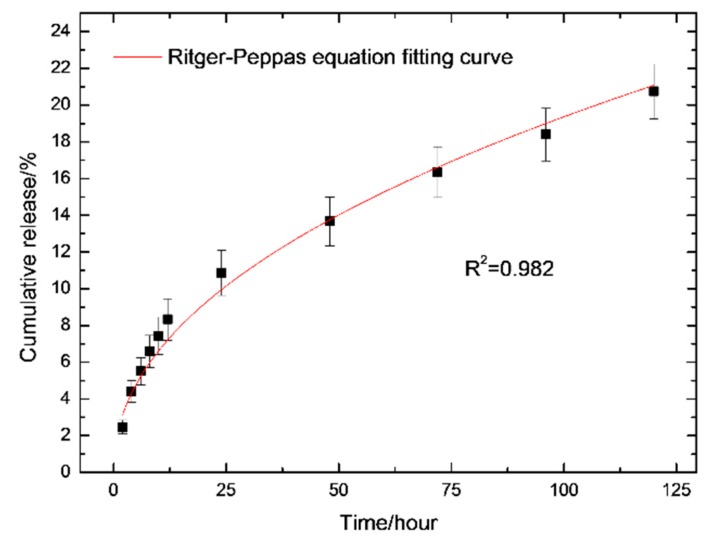
Release of RB from the prepared hydrogel with curve fitting corresponding to the Ritger–Peppas equation. The adjust R-square is 0.982, indicating that the curve is reliable.

It was found the drug release reaches its steady state 48 h (Figure 6) after incubation with PBS solution. The value for n was obtained as 0.47 ± 0.02 after fitting curves to the data based on the Ritger–Peppas equation (Figure 7). This means that the drug release from hydrogel follows an anomalous transport mechanism [50], and RB can be completely released from the hydrogel after 3382 h of incubation with PBS solution, based on Equation (3).

### 3.7. MTT Assay

**Figure 8:**
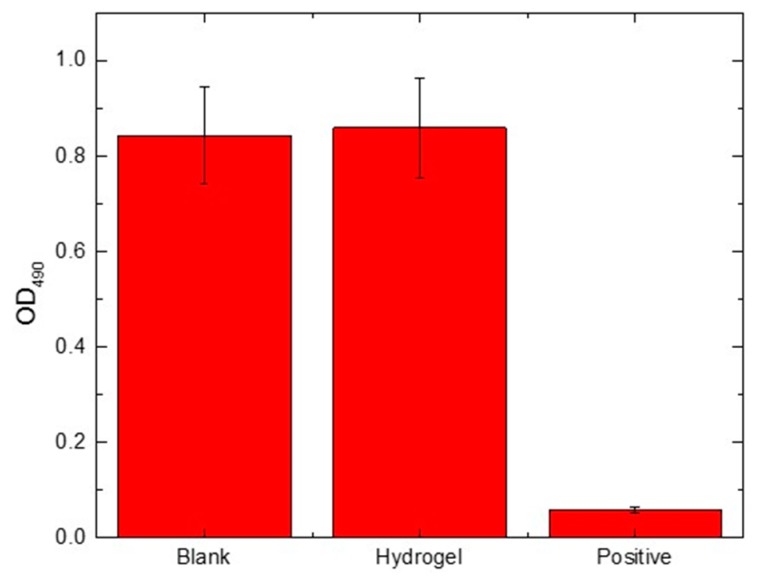
Viability of human embryonic kidney (HEK) 293 cells cultured for 24 h.

The in vitro cytotoxicity of the prepared hydrogel was evaluated using the MTT assay of HEK 293 cells. Yellow MTT would be converted to purple formazan by the mitochondrial succinate dehydrogenases in the living cell, and the amount of formazan is considered to be proportional to the number of living cells [51]. The amount of the formazan could be quantified by a microplate reader after dissolving in DMSO. The OD values of the three groups—blank, experimental, and positive control—at 490 nm were demonstrated in Figure 8. The viability of the cells was determined based on the following formula:

(4) $$Cell\;viability\;\left(\%\right)=\frac{A}{B}\times 100\%,$$

where *A* is the OD value of the experimental group, and *B* is the OD value of the blank group.

The cell viability of the experimental groups was 101.9 ± 14.5%, and the value was not significantly different from that of blank, indicating that the prepared hydrogel is non-cytotoxic.

**Figure 9:**
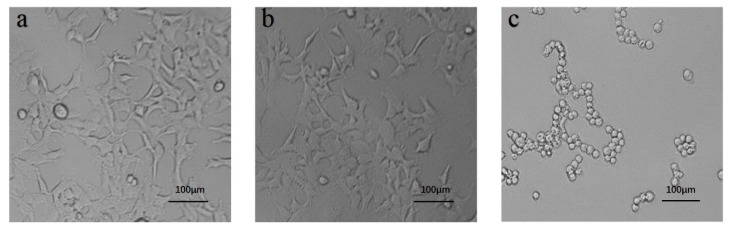
The representative cell morphology of 3-(4,5-dimethythiazol-2-yl)-2,5-diphenyl tetrazolium bromide (MTT) assay at 24 h. (**a**) Blank, (**b**) experimental group, and (**c**) positive control (scale bar 100 μm).

Figure 9 presents the cell morphology of blank, experimental, and positive control in a 96-well plate observed under an inverted microscope. In both blank and experimental groups, cells appeared elongated and spindle shaped with the pseudopodium stretched out, which is similar to the HEK 293 morphology (CRL-1573) from the American Type Culture Collection (ATCC). These data indicated that the prepared hydrogel has no adverse influence on the growth of HEK 293 cells. In contrast, the cells in positive controls adhered together and exhibited a rounded cell body, which is prone to apoptosis.

### 3.8. Subcutaneous Implantation of PEGDA/SF Hydrogel

**Figure 10:**
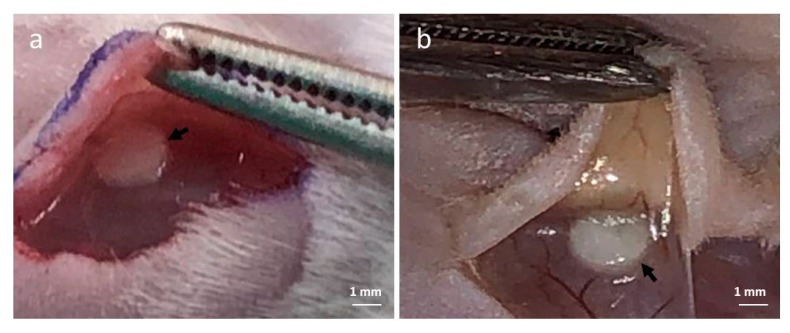
(**a**) Representative photograph of the poly(ethylene glycol) diacrylate (PEGDA)/SF hydrogel at the time of subcutaneous implantation. (**b**) Tissue appearance of mice subcutis three days after implantation of the PEGDA/SF hydrogel. (Arrow: PEGDA/SF hydrogel).

In order to evaluate the in vivo compatibility of the PEGDA/SF hydrogel, the subcutaneous implantation of the hydrogels was performed, and a subcutaneous pocket on the dorsum of mice was created, and the samples were implanted into the pocket (Figure 10a). Figure 10b shows the tissue appearance of skin back sides following the implantation of hydrogels after three days. The implantation of the PEGDA/SF hydrogel did not affect the vascularity in the subcutis at all. No problematic and severe inflammatory response to the implanted hydrogels was observed.

### 3.9. Histological Analysis

**Figure 11:**
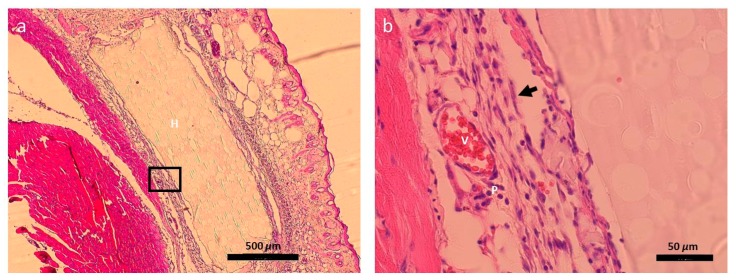
(**a**) Histological sections of mice subcutis three days after the implantation of PEGDA/SF hydrogel. The hydrogel embedded within the tissue exhibited a porous structure, which is consistent with SEM observation. The black box is the zone shown in (**b**) (H: Hydrogel; V: Blood vessel; P: Plasma cell; Arrow: Fibroblast).

The tissue slides were H&E stained to access cellularity. It was observed that the implanted hydrogel was within the existing tissue and maintained its structural integrity (Figure 11a). In addition, the PEGDA/SF hydrogel was adhered by a layer of granulation tissue, which consists of blood vessels, fibroblasts, and plasma cells [52]. The microscopic evaluation demonstrated that there was no obvious inflammation after implantation of the hydrogel into the mice for 3 days, which was consistent with its macroscopic image in Figure 10b. In a word, the PEGDA/SF hydrogel possess excellent biocompatibility in vivo, which makes it an ideal material for drug delivery.

## 4. Discussion

**Scheme 1:**
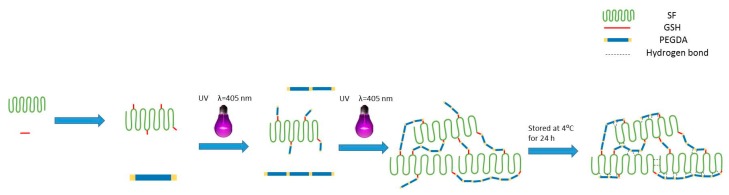
Schematic of PEGDA/SF hydrogel preparation procedure: SF was modified by GSH at first, then PEGDA was grafted to GSH-SF under UV light exposition. With an increase in the exposure time to UV light, the material undergoes a phase change from liquid to hydrogel due to the crosslinking of PEGDA. Then, the prepared PEGDA/SF hydrogels were stored in the refrigerator at 4 °C for hydrogen bond formation.

We have observed that UV illumination can induce the self-gelation of PEGDA molecules (Supplementary Material, Figure S2a). Accompanied by FTIR results which confirmed the completion of the thiol–ene reaction between GSH-SF and PEGDA (Figure 4a), we proposed the mechanism of the PEGDA/SF gelation process (Scheme 1). The formation of the prepared hydrogel shall have two distinct but integrated stages. First, thiol-grafted SF molecules served as a core to interact with PEGDA through the thiol–ene click reaction rapidly [53]. Then, PEGDA molecules crosslinked to each other under UV light illumination surrounded SF molecules and adopted the mechanism of PEGDA self-gelation to form a gel structure [54]. Meanwhile, the low-temperature step increases the number of hydrogen bonds, which strengthen the PEGDA/SF hybrid networks [41,42].

## 5. Conclusions

Here, we developed a new method of PEGDA/SF hydrogel preparation through a thiol–ene click reaction without the use of a photoinitiator. First, 405 nm blue light was used for photocrosslinking, which may improve the implementation of the thiol–ene click reaction in biomedical applications requiring low, cytocompatible doses of light. The thiol–ene click reaction played a key role in the overall gelation process, which installed PEGDA molecules onto SF molecules. Then, PEGDA-installed SF molecules served as focal points or core, participating in the build-up of hydrogel networks. The prepared hydrogel could be obtained in a short and controllable gelation time. Further, the compressive strength of the prepared PEGDA/SF hydrogel was improved by introducing a prior cooling step that can be utilized in the control of hydrogel property.

In this study, FTIR was used to determine the successful grafting of PEGDA to SF and analyze the secondary structure of the prepared PEGDA/SF hydrogel. The compressive test results revealed that hydrogen bonding plays a crucial role in influencing the mechanical properties of the prepared PEGDA/SF hydrogel. The SEM photos showed that the prepared hydrogel is a porous structure, indicating that the PEGDA/SF hydrogel could be used to load drugs or cells. Drug release and swelling ratio experiments confirmed that PEGDA/SF hydrogel possesses the potential to be a drug delivery system. The MTT assay results demonstrated that the prepared PEGDA/SF hydrogel was non-cytotoxic to HEK 293 cells. In addition, the subcutaneous implantation of PEGDA/SF hydrogel did not show any acute inflammatory effects in mice, revealing that the PEGDA/SF hydrogel possessed favorable biocompatibility. We expected that this thiol–ene click reaction-centered hydrogel fabrication method is efficient in SF-based hydrogel preparation for biomedical application.

## Figures and Tables

**Table 1:** The diameter of pores present.

Sample (UV Light Treated Time)	The Pore’s Average Diameter
1 min	16.91 ± 7.26 μm
2 min	10.61 ± 4.35 μm
3 min	7.37 ± 5.52 μm

## Supplementary Materials

The following are available online at https://www.mdpi.com/2073-4360/11/12/2102/s1, Figure S1. The linear calibration curve was acquired by plotting a UV-Vis absorbance of 3 mL of a series of known concentrations of rhodamine B in PBS solution. Figure S2. a. Morphology of PEGDA hydrogel prepared after 30 min of UV light illumination (405 nm) of PEGDA and DI H_2_O mixture at a 1:1 ratio (*v*/*v*); b. SEM image of the prepared PEGDA hydrogel displaying a porous free structure. Figure S3. Pore size distribution of the prepared PEGDA/SF hydrogels.

## References

[1] Ribeiro M., Fernandes M.H., Beppu M.M., Monteiro F.J., Ferraz M.P. (2018). Silk fibroin/nanohydroxyapatite hydrogels for promoted bioactivity and osteoblastic proliferation and differentiation of human bone marrow stromal cells. Mater. Sci. Eng. C.

[2] Mao B., Liu C., Zheng W., Li X., Ge R., Shen H., Guo X., Lian Q., Shen X., Li C. (2018). Cyclic cRGDfk peptide and chlorin e6 functionalized silk fibroin nanoparticles for targeted drug delivery and photodynamic therapy. Biomaterials.

[3] Wang D., Liu H., Fan Y. (2015). Silk fibroin for vascular regeneration. Microsc. Res. Tech..

[4] Ma D., Wang Y., Dai W. (2018). Silk fibroin-based biomaterials for musculoskeletal tissue engineering. Mater. Sci. Eng. C.

[5] Yang N., Qi P., Ren J., Yu H., Ling S. (2019). Polyvinyl alcohol/silk fibroin/borax hydrogel ionotronics: A highly stretchable, self-healable, and biocompatible sensing platform. ACS Appl. Mater. Interfaces.

[6] Dong T., Mi R.X., Wu M., Zhong N.P., Zhao X., Chen X., Shao Z.S. (2019). The regenerated silk fibroin hydrogel with designed architecture bioprinted by its microhydrogel. J. Mater. Chem. B.

[7] Lai Y., Hu Y. (2018). Probing the swelling-dependent mechanical and transport properties of polyacrylamide hydrogels through AFM-based dynamic nanoindentation. Soft Matter.

[8] Cao H., Duan Y., Lin Q., Yang Y., Shao Z. (2019). Dual-loaded, long-term sustained drug releasing and thixotropic hydrogel for localized chemotherapy of cancer. Biomater. Sci..

[9] Dosh R.H., Jordan-Mahy N., Sammon C., Maitre C.L.L. (2019). Use of L-pNIPAM hydrogel as a 3D-scaffold for intestinal crypts and stem cell tissue engineering. Biomater. Sci..

[10] Joo C., Hayan J., Jeong S., Joaquim O., Rui R., Gilson K. (2018). Biofunctionalized lysophosphatidic acid/silk fibroin film for cornea endothelial cell regeneration. Nanomaterials.

[11] Mcgill M., Coburn J.M., Partlow B.P., Mu X., Kaplan D.L. (2017). Molecular and macro-scale analysis of enzyme-crosslinked silk hydrogels for rational biomaterial design. Acta Biomater..

[12] Luo K., Yang Y., Shao Z. (2016). Physically crosslinked biocompatible silk-fibroin-based hydrogels with high mechanical performance. Adv. Funct. Mater..

[13] Pacelli S., Paolicelli P., Avitabile M., Varani G., Casadei M.A. (2018). Design of a tunable nanocomposite double network hydrogel based on gellan gum for drug delivery applications. Eur. Polym. J..

[14] Floren M.L., Spilimbergo S., Motta A., Migliaresi C. (2012). Carbon dioxide induced silk protein gelation for biomedical applications. Biomacromolecules.

[15] Wang X., Kluge J.A., Leisk G.G., Kaplan D.L. (2008). Sonication-induced gelation of silk fibroin for cell encapsulation. Biomaterials.

[16] Hari K., Alaeddin A., Haris Z., Elaine S., Bipin P., Harsha J., Morshed K. (2017). Evaluation of polyethylene glycol diacrylate-polycaprolactone scaffolds for tissue engineering applications. J. Funct. Biomater..

[17] Schesny M.K., Monaghan M., Bindermann A.H., Freund D., Seifert M., Eble J.A., Vogel S., Gawaz M.P., Hinderer S., Schenke-Layland K. (2014). Preserved bioactivity and tunable release of a SDF1-GPVI bi-specific protein using photo-crosslinked PEGda hydrogels. Biomaterials.

[18] Xia B., Jiang Z., Debroy D., Li D., Oakey J. (2017). Cytocompatible cell encapsulation via hydrogel photopolymerization in microfluidic emulsion droplets. Biomicrofluidics.

[19] Sullivan J.P.A., Stevenson P., Johansson K., Parker K. (2014). Rapid room-temperature-curing monomer platform. Paint & Coatings Industry.

[20] (2019). 1- Hydroxycyclohexyl Phenyl Ketone.

[21] (2019). 2,4,6-Trimethylbenzoyl-Diphenylphosphine Oxide.

[22] (2019). Phenylbis(2,4,6-Trimethylbenzoyl)Phosphine Oxide.

[23] (2019). 2-Hydroxy-4′-(2-Hydroxyethoxy)-2-Methylpropiophenone.

[24] Chan-Park M.B., Zhu A., Chu S.L., Lim H.C. (2004). Argon-plasma-assisted graft polymerization of thick hydrogels with controllable water swelling on chronoflex. J. Adhes. Sci. Technol..

[25] Li H., Ma T., Zhang M., Zhu J., Liu J., Tan F. (2018). Fabrication of sulphonated poly(ethylene glycol)-diacrylate hydrogel as a bone grafting scaffold. J. Mater. Sci. Mater. Med..

[26] Nelson D., Cox M. (2013). Lehninger Principles of Biochemistry.

[27] Ryu S., Kim H.H., Park Y.H., Lin C.C., Um I.C., Ki C.S. (2016). Dual mode gelation behavior of silk fibroin microgel embedded poly(ethylene glycol) hydrogels. J. Mater. Chem. B.

[28] (2018). Cis-5-Norbornene-endo-2,3-Dicarboxylic Anhydride.

[29] Gregoritza M., Abstiens K., Graf M. (2018). Fabrication of antibody-loaded microgels using microfluidics and thiol-ene photoclick chemistry. Eur. J. Pharm. Biopharm..

[30] Mckenas C.G., Fehr J.M., Donley C.L., Lockett M.R. (2016). Thiol-ene modified amorphous carbon substrates: Surface patterning and chemically modified electrode preparation. Langmuir.

[31] (2019). 2,2-Dimethoxy-2-Phenylacetophenone.

[32] (2019). Diphenyl(2,4,6-Trimethylbenzoyl)Phosphine Oxide.

[33] (2019). Benzophenone.

[34] (2015). Isobutyronitrile.

[35] Zhang X., Bao H., Donley C., Liang J., Xu S. (2018). Thiolation and characterization of regenerated Bombyx mori silk fibroin films with reduced glutathione. BMC Chem..

[36] Hu W., Feng X., Liu X., Dai S., Zeng W., Jiang Q., Chen B., Quan C., Sun K., Zhang C. (2016). Poly(γ-glutamic acid) modulates the properties of poly(ethylene glycol) hydrogel for biomedical applications. J. Biomater. Sci. Polym. Ed..

[37] Gong C., Shan M., Li B., Wu G. (2017). Injectable dual redox responsive diselenide-containing polyethylene glycol hydrogel. J. Biomed. Mater. Res. Part A.

[38] Zhu L., Qiu J., Sakai E. (2017). A high modulus hydrogel obtained from hydrogen bond reconstruction and its application in vibration damper. RSC Adv..

[39] Wang H., Zhu H., Fu W., Zhang Y., Xu B., Gao F., Cao Z., Liu W. (2017). A high strength self-healable antibacterial and anti-inflammatory supramolecular polymer hydrogel. Macromol. Rapid Commun..

[40] Guo M., Pitet L.M., Wyss H.M., Vos M., Dankers P.Y.W., Meijer E.W. (2014). Tough stimuli-responsive supramolecular hydrogels with hydrogen-bonding network junctions. J. Am. Chem. Soc..

[41] Cai J., Zhang L., Chang C., Cheng G., Chen X., Chu B. (2007). Hydrogen-bond-induced inclusion complex in aqueous cellulose/LiOH/urea solution at low temperature. Chemphyschem.

[42] Xia S., Song S., Jia F., Gao G. (2019). A flexible, adhesive and self-healable hydrogel-based wearable strain sensor for human motion and physiological signal monitoring. J. Mater. Chem. B.

[43] Tytgat L., Vagenende M., Declercq H., Martins J.C., Vlierberghe S.V. (2018). Synergistic effect of κ-carrageenan and gelatin blends towards adipose tissue engineering. Carbohydr. Polym..

[44] Carbinatto F.M., de Castro A.D., Evangelista R.C., Cury B.S.F. (2014). Insights into the swelling process and drug release mechanisms from cross-linked pectin/high amylose starch matrices. Asian J. Pharm. Sci..

[45] Tan H.P., Tan H.P. (2017). Base-pairing hydrogel. Medical Biodegradable Hydrogel Material.

[46] Liu L., Wen H., Rao Z., Zhu C., Tao S. (2017). Preparation and characterization of chitosan—Collagen peptide/oxidized konjac glucomannan hydrogel. Int. J. Biol. Macromol..

[47] Zarrintaj P., Urbanska A.M., Gholizadeh S.S., Goodarzi V., Saeb M.R., Mozafari M. (2018). A facile route to the synthesis of anilinic electroactive colloidal hydrogels for neural tissue engineering applications. J. Colloid Interface Sci..

[48] Shin J., Choi S., Kim J.H., Cho J.H., Cho S.W. (2019). Tissue tapes—Phenolic hyaluronic acid hydrogel patches for off-the-Shelf therapy. Adv. Funct. Mater..

[49] Lucht L.M., Peppas N.A. (1987). Transport of penetrants in the macromolecular structure of coals. V. anomalous transport in pretreated coal particles. J. Appl. Polym. Sci..

[50] Vermonden T., Censi R., Hennink W.E. (2012). Hydrogels for protein delivery. Chem. Rev..

[51] Maitlo I., Ali S., Akram M.Y., Shehzad F.K., Nie J. (2017). Binary phase solid-state photopolymerization of acrylates: Design, characterization and biomineralization of 3D scaffolds for tissue engineering. Front. Mater. Sci..

[52] LibrePathology.

[53] Xu K., Fu Y., Chung W., Zheng X., Cui Y., Hsu I.C., Kao W.J. (2012). Thiol-ene-based biological/synthetic hybrid biomatrix for 3D living cell culture. ACTA Biomater..

[54] Yao H., Wang J.Q., Mi S.L. (2017). Photo processing for biomedical hydrogels design and functionality: A review. Polymers.

