# Supplementary material for: Thiol–Ene Click Reaction Initiated Rapid Gelation of PEGDA/Silk Fibroin Hydrogels
Source: Polymers (Basel). 2019 Dec 14;11(12):2102. doi: 10.3390/polym11122102 (PMC6960922; doi:10.3390/polym11122102)
Supplement: Supplementary file 1 [file polymers-11-02102-s001.pdf]

Supplementary Matherails

Thiol–Ene Click Reaction Initiated Rapid Gelation of PEGDA/Silk Fibroin Hydrogels

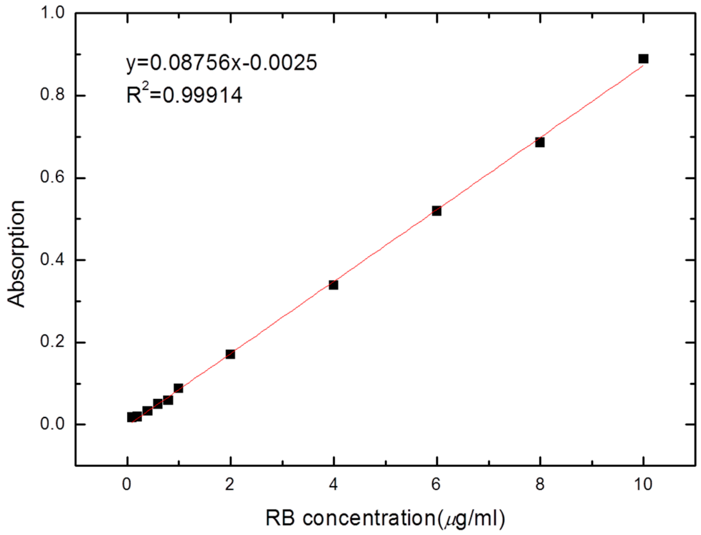

Figure S1 The linear calibration curve was acquired by plotting UV-Vis absorbance of 3 mL of a series of known concentrations of rhodamine B in PBS solution.

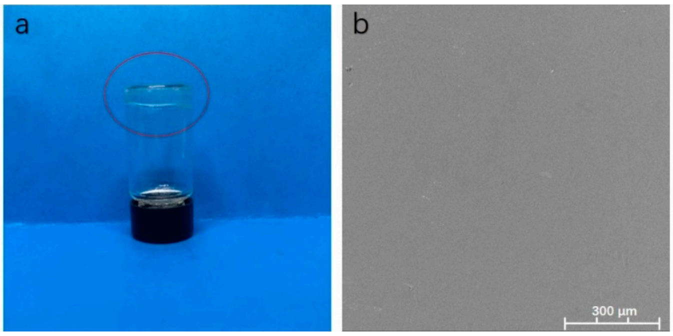

Figure S2 a. Morphology of PEGDA hydrogel prepared after 30 minutes UV light illumination (405 nm) of PEGDA and DI H_2O mixture at a 1:1 ratio (v/v); b. SEM image of the prepared PEGDA hydrogel displaying a porous free structure.

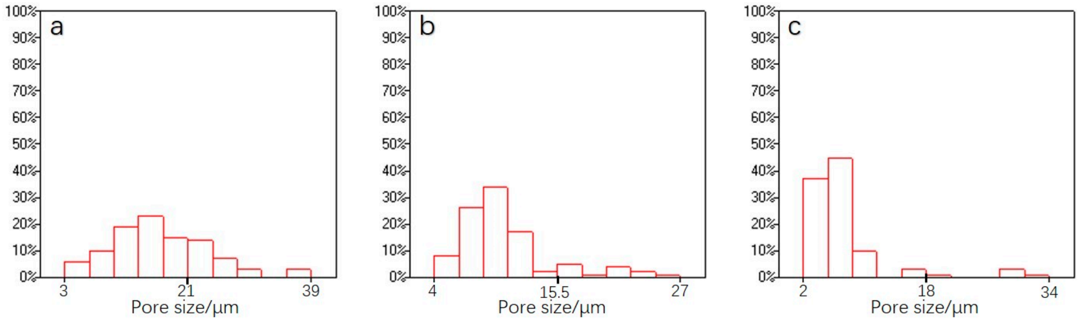

Figure S3 Pore size distribution of the prepared PEGDA/SF hydrogels. For each group, 100 pores from the SEM photo were chose, and the pore size was computed with software "Nano Measurer" (version, 1.2.5). a, b, c were the pore size distribution of hydrogel prepared by UV light illumination for 1 minute, 2 minutes, and 3 minutes respectively. It was found that the pore size was reduced as the UV-light illumination time increase.
